# Supplementary material for: Improved metagenome assemblies and taxonomic binning using long-read circular consensus sequence data
Source: Sci Rep. 2016 May 9;6:25373. doi: 10.1038/srep25373 (PMC4860591; doi:10.1038/srep25373)
Supplement: Supplementary Information [file srep25373-s1.pdf]

Improved metagenome assemblies and taxonomic binning using long-read circular consensus sequence data

J. A. Frank[1], Y. Pan[2], A. Tooming-Klunderud[3], V.G.H. Eijsink[1], A.C. McHardy[2], A. J. Nederbragt[3], P.B. Pope[1*]

1. Department of Chemistry, Biotechnology and Food Science, Norwegian University of Life Sciences, Ås, 1432 NORWAY.
2. Computational Biology of Infection Research, Helmholtz Centre for Infection Research, Inhoffenstraβe 7, 38124 Braunschweig.GERMANY.
3. University of Oslo, Department of Biosciences, Centre for Ecological and Evolutionary Synthesis, Blindern, 0316 NORWAY.

***Corresponding Author:** Phillip B. Pope
Department of Chemistry, Biotechnology and Food Science
Norwegian University of Life Sciences
Post Office Box 5003
1432, Ås
Norway
Phone: +47 6496 6232
Email: phil.pope@nmbu.no

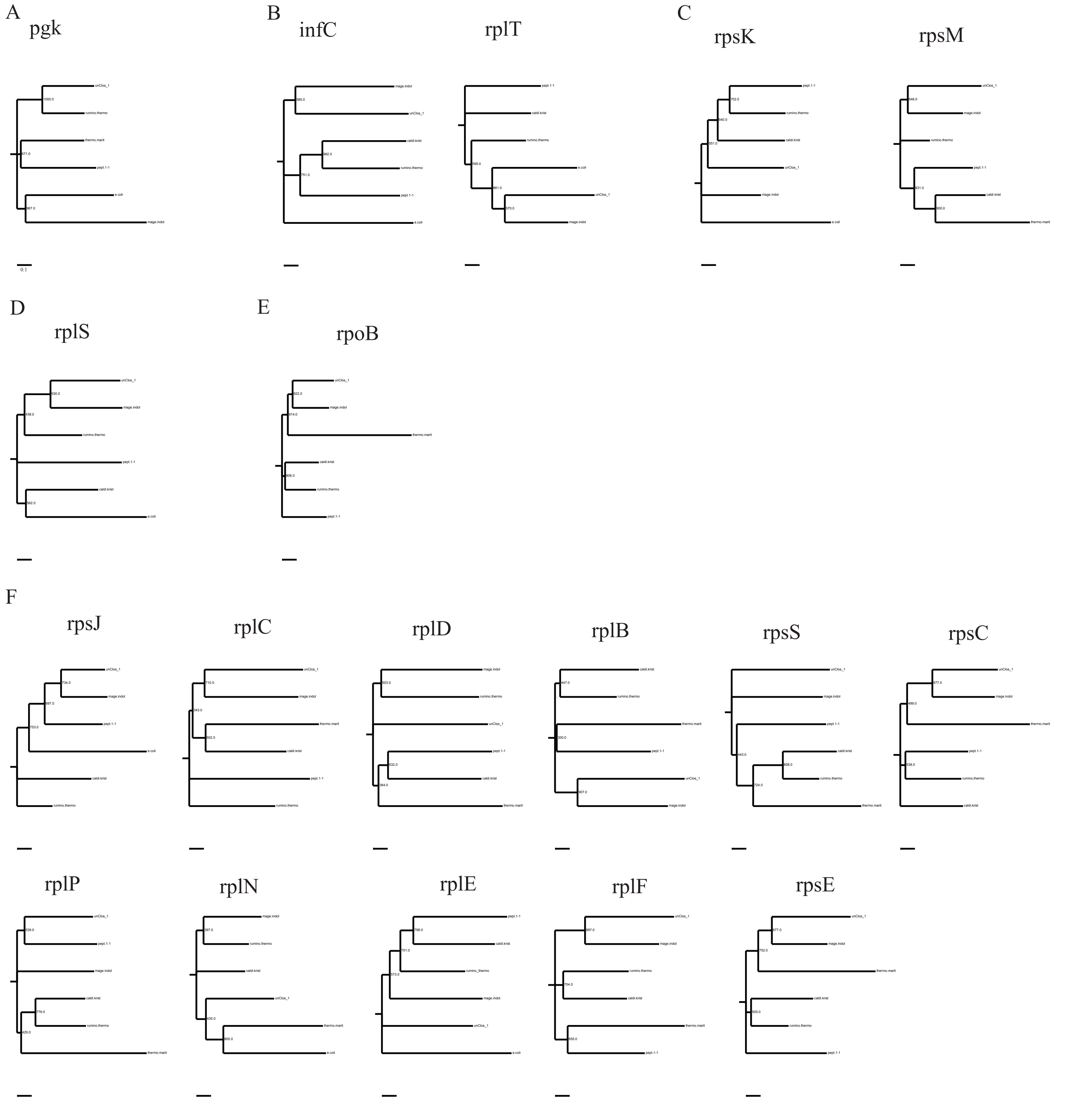

Figure S1. Marker gene phylogenies from contigs used as training data for unClos_1 phylogenomic binning. The mid-pointed rooted trees are organized by source contig to evaluate and compare the phylogeny of the marker genes that were encoded within contigs used for unClos_1 PhyloPythiaS+ training data. The genes identified are the phosphoglycerate kinase (pgk), translation initiation factor IF-3 (infC), 50S ribosomal protein L20 (rplT), 30S ribosomal protein S11 (rpsK), 30S ribosomal protein S13 (rpsM), 50S ribosomal protein L19 (rplS), DNA-directed RNA polymerase beta subunit (rpoB), 30S ribosomal protein S10 (rpsJ), 30S ribosomal protein S3 (rpsC), 50S ribosomal protein L4 (rplD), 50S ribosomal protein L2 (rplB), 30S ribosomal protein S19 (rpsS), 30S ribosomal protein S3 (rpsC), 50S ribosomal protein L16 (rplP), 50S ribosomal protein L14 (rplN), 50S ribosomal protein L5 (rplE), 50S ribosomal protein L6 (rplF), and 30S ribosomal protein S5 (rpsE). The reference organisms used are *Mageeibacillus indolicus* (mage.indol), *Ruminiclostridium thermocellum* (rumino.thermo), *Caldicellulosiruptor kristjanssonii* (caldi.krist), *Peptoniphilus sp.* 1-1 (pept.1-1), and *E. coli* (e.coli) and *Thermotoga maritima* (thermos.marit) as outliers. Divergence bars below each tree are 0.1.

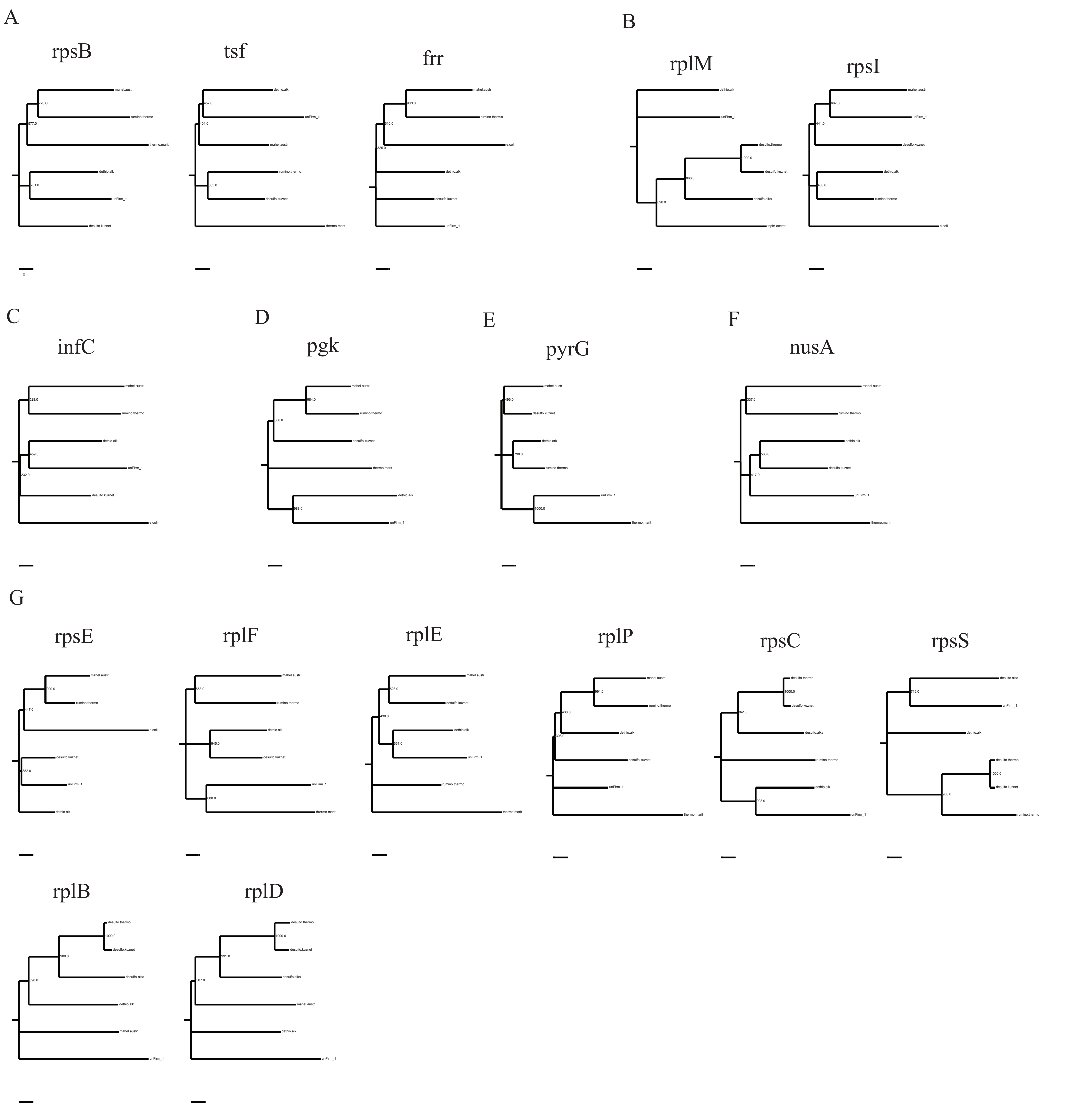

Figure S2. Marker gene phylogenies from contigs used as training data for unFirm_1 phylogenomic binning. The mid-pointed rooted trees are organized by source contig to evaluate and compare the phylogeny of the marker genes that were encoded within contigs used for unFirm_1 PhyloPythiaS+ training data. The genes identified are the 30S ribosomal protein S2 (rpsB), elongation factor Ts (tsf), 30S ribosomal protein S9 (rpsI), translation initiation factor IF-3 (infC), phosphoglycerate kinase (pgk), CTP synthase (pyrG), transcription termination/antitermination protein (nusA), 30S ribosomal protein S5 (rpsE), 50S ribosomal protein L6 (rplF), 50S ribosomal protein L5 (rplE), 50S ribosomal protein L16 (rplP), 30S ribosomal protein S3 (rpsC), 50S ribosomal protein L2 (rplB), and 50S ribosomal protein L4 (rplD). The reference organisms used are *Mahella australiense* (mahel.austr), *Ruminiclostridium thermocellum* (rumino.thermo), *Dethiobacter alkaphilus* (dethio.alk), *Desulfotomaculum kuznetsovii* (desulfo.kuznet), and *E. coli* (e.coli) and *Thermotoga maritima* (thermos.marit) as outliers. Divergence bars below each tree are 0.1.
